# Association Between Anterior Rhinoscopic/Endoscopic Assessment of Internal Nasal Valve and Various Nasal Deformities in the Rural Population of Vidarbha Region of India: A Cross-Sectional Study

**DOI:** 10.7759/cureus.35682

**Published:** 2023-03-02

**Authors:** Senu Sunnychan, Prasad Deshmukh

**Affiliations:** 1 Otolaryngology - Head and Neck Surgery, Jawaharlal Nehru Medical College, Datta Meghe Institute of Higher Education and Research, Wardha, IND

**Keywords:** diagnostic nasal endoscopy, nasal resistance, deviated nasal septum, mladina classification, internal nasal valve

## Abstract

Background

The nasal valves constitute the majority of the nasal cavity's resistance. Any reduction in this already narrow area can cause a significant reduction in the nasal airflow. In this present study, the aim was to do an endoscopic assessment of the internal nasal valve (INV) in patients with various nasal septal deviations, with or without external nasal deformity. We measured endoscopically the INV in various nasal deformities and derived its association with the INV on anterior rhinoscopy and endoscopic assessment.

Method

We included 75 patients in the study who were analyzed for angle and grade of the INV by anterior rhinoscopic examination and Hopkins rod zero-degree nasal endoscope (Karl Storz SE & Co., Tuttlingen, Germany). Nasal septal deviations were also studied with respect to the Mladina classification. Correlation between various nasal septal deviations with the INV was done. Since studies addressing the classification of INV are not available in the literature, for the purpose of simplification of observation of INV angle, (normal range 9-15 degrees), subjective stratification was made in the study, i.e. below 9, 9-15, and more than 15 degrees for the sake of knowing the underlying cause and its relationship.

Result

An anterior rhinoscopic examination was performed on 75 patients. INV Grade 1 was the most common, with 18 patients of (69.2%), 15 patients of DNS with caudal dislocation (55.6%), five patients of DNS with spur (38.5%), and four patients of DNS with external nasal deformity (50%). The next frequently observed Grade of INV on anterior rhinoscopy examination was Grade 2, in 11 patients of DNS with caudal dislocation (40.7%), four patients of DNS with spur (30.8%), and three patients of DNS with external deformity (37.5%), which was statistically significant in our study. In the majority of patients with all types of nasal septal deviations with or without external nasal deformity, INV (angle) of less than 9 degrees was noted, which was statistically significant. A linear relationship, ie., Grade 0 INV in Type I, Grade 1 INV in types II, III, IV, and V, and Grade 2 in Type VII was observed. Our study is on par with the literature questioning the dogma of the normal angle of INV being 9-15 degrees.

Conclusion

We were able to establish a positive and complimentary role of anterior rhinoscopic and endoscopic assessment of INV. The proposed novel classification of the angle of INV by endoscopic assessment gives a better insight into the association of INV with various nasal septal deformities with or without external nasal septal deviation.

## Introduction

Nasal airway resistance comprises more than 50% of total airway resistance and plays a crucial role in nasal breathing [1]. There is always a pressure gradient in the airway from a high-pressure to a low-pressure area, which causes nasal airflow [2]. The sympathetic nervous system significantly affects nasal resistance due to its action on the erectile tissue (venous) of the inferior and middle turbinate, causing a difference in the engorgement of the capacitance vessels of the nasal mucosa [3].

The most common etiologies of unilateral nasal obstruction are nasal septal deviations (NSDs), inferior turbinate hypertrophy, unilateral sinusitis, antrochoanal polyps, congenital choanal atresia, trauma, rhinoscleroma, and in the case of bilateral nasal obstruction, acute rhinitis, adenoid hypertrophy, trauma (septal hematoma), rhinitis medicamentosa, and sino-nasal malignancies are the most common etiologies [3,4]. Internal nasal valve (INV) obstruction is frequently overlooked although it is one of the common causes of structural nasal obstruction [5].

NSD classifications are complex, and this complexity is reciprocated in different populations [6]. According to research, the prevalence of NSD ranges from 5% to 85% among different age groups [6,7].

The nasal valve area comprises the septum, the upper lateral cartilage (ULC)'s caudal border, the head of the inferior turbinate head, the pyriform aperture, and the surrounding tissues [8]. More than two-thirds of the resistance the nose produces comes from this location [9,10]. The INV contributes significantly to nasal obstruction and is crucial for middle ear and sinus ventilation [11]. The pathophysiology of the sinuses and middle ear is significantly impacted by any further narrowing of the INV. A static structural variation due to a high NSD, enlarged turbinates, and dynamic collapse due to weakness in the ULC/lateral wall of the nose can cause abnormality during inspiration leading to INV obstruction [12]. Although they are separate entities, static and dynamic INV collapses can coexist [13]. The present work aims to study endoscopically the INV in various NSDs with/without external nasal deformities and draw its association with INV on anterior rhinoscopy and endoscopic assessment.

## Materials and methods

This is a cross-sectional study conducted on patients having NSD with or without external nasal deformity visiting the outpatient department (OPD), ENT inpatient department (IPD), and casualty of Acharya Vinoba Bhave Hospital, Wardha, Maharashtra, India from December 2020 to December 2022. The inclusion and exclusion criteria are listed in Table 1.

**Table 1:** Inclusion and exclusion criteria for the study

Serial no.	Inclusion criteria
1	All the patients with nasal septal deviation and external nasal deformities with or without allergy in the age group of 15-60 years
	Exclusion criteria
1	All the patients of nasal septal deformity with or without external nasal deformities below 15 years and above 60 years of age.
2	Patients with other obstructive lesions of upper and lower airway.
3	Patients with other granulomatous and malignant lesions of nasal cavity.
4	Patients with cardiovascular, neurological, and respiratory disorders.

The study included 75 patients, in the age group of 15-60 years of age, who underwent anterior rhinoscopic examination with particular importance given to the INV and graded based on a horizontal line at the level of the head of the inferior turbinate as done by Patel et al. [5]. Grade 0 indicates an evident middle turbinate head. Grade 1 indicates a slightly occluded middle turbinate. The middle turbinate is not visible in Grade 2 (maximum grade). Diagnostic nasal endoscopy (DNE) was done with a rigid endoscope, Hopkins rod zero degree nasal endoscope (4.0 mm) (Karl Storz SE & Co., Tuttlingen, Germany) where the angle of the INV was measured in each nostril at rest, at the level of the head of the inferior turbinate. DNE video was processed in ScopyDoc 7.0.2 software (Medsynaptic Pvt. Ltd., Pune, India), and the exact angle of the INV was measured. NSDs were also studied with respect to the Mladina classification [14]. Since studies addressing the classification of INV are not available in the literature, for the purpose of simplification of observation of INV angle (normal range 9-15 degrees), subjective stratification was made in the study, i.e. below 9, 9-15, and more than 15 degrees for the sake of knowing the underlying cause and its relationship. The study employed descriptive statistics including frequency and percentage along with Pearson chi-square test to determine the influence of one variable on the other. A significance level of p<0.05 was considered for statistical significance.

Ethical clearance

All procedures performed in this study involving human participants were in accordance with the ethical standards of the Internal Institutional Ethics Committee, Datta Meghe Institute of Higher Education and Research, Maharashtra, India (approval number: DMIMS(DU)/IEC/2020-21/9328, dated December 22, 2020). The approval has been granted on the assumption that the proposed work will be carried out in accordance with the ethical guidelines prescribed by Central Ethics Committee on Human Research (CECHR). 

## Results

Out of the 75 patients with NSDs with or without external nasal deformity, 26 patients (34.7%) had deviated nasal septum (DNS), 27 patients (36.0%) had DNS with caudal dislocation, and 13 patients (17.3%) had DNS and spur. We observed DNS with external deformity in eight (10.7%) patients, whereas a lone patient had DNS with septal thickening.

**Figure 1:**
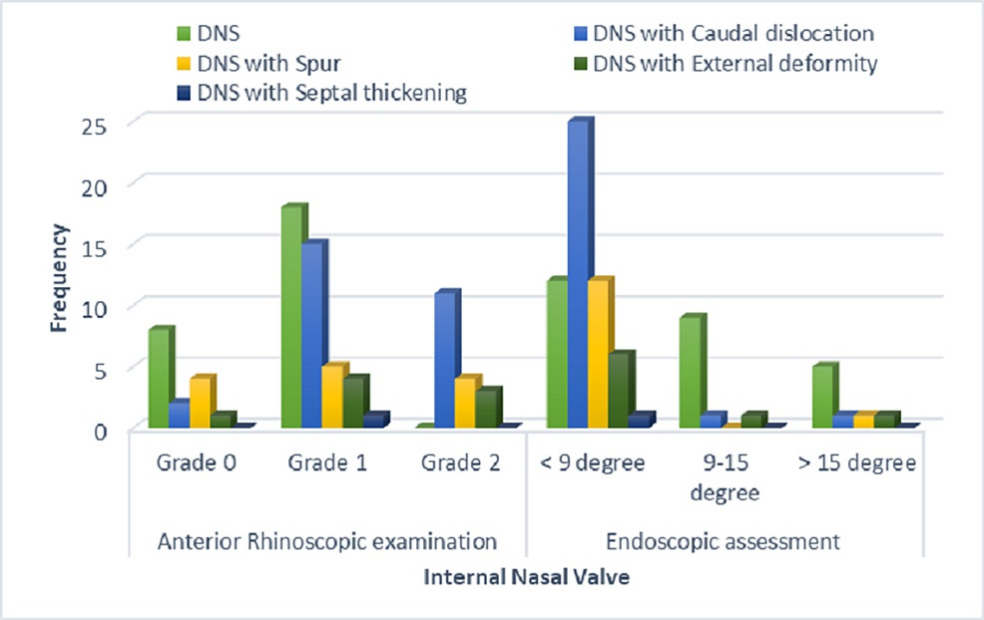
Association of nasal septal deviation and external deformities with internal nasal valve DNS: deviated nasal septum

Table 2 and Figure 1 show the association of NSD and external deformities with the INV on anterior rhinoscopic examination. INV Grade 1 was most common with DNS (69.2%), DNS with caudal dislocation (55.6%), DNS with spur (38.5%), and DNS with external nasal deformity (50%). The next frequently observed Grade of INV was Grade 2, in 11 patients with DNS with caudal dislocation (40.7%), four patients with DNS with spur (30.8%), and three patients with DNS with external deformity (37.5%), which was statistically significant. (X² = 18.52; p= .017) 

**Table 2:** Association of nasal septal deviation and external deformities with internal nasal valve (anterior rhinoscopic examination) DNS: deviated nasal septum

	Anterior Rhinoscopic Examination of Internal Nasal Valve			Chi-square value
	Grade 0, n(%)	Grade 1, n (%)	Grade 2, (%)	
DNS (26)	8 (30.77%)	18 (69.2%)	0 (0%)	X^2^ = 18.52 p=.017
DNS with caudal dislocation (27)	2 (7.41%)	15 (55.6%)	11 (40.7%)	
DNS with spur (13)	4 (30.77%)	5 (38.5%)	4 (30.8%)	
DNS with external deformity (8)	1 (12.5%)	4 (50%)	3 (37.5%)	
DNS with septal thickening (1)	0 (0.0%)	1 (100%)	0 (0.0%)	

Table 3 and Figure 1 show the association of NSD and external deformities with INV angle on endoscopic assessment. It was observed that the INV angle in the endoscopic examination was 9-15 degrees in nine (34.6%) DNS patients, one DNS patient with caudal dislocation (3.7%), and one DNS patient with external deformity (12.5%). In 12 (46.2%) DNS patients, INV angle < 9 degrees was observed; 25 DNS patients with caudal dislocation (92.6%), 12 DNS patients with spur (92.3%), six DNS patients with external deformity (75%), and one DNS patient with septal thickening (100%). Thus, in the majority of patients with all types of NSDs with or without external nasal deformity, an INV (angle) < 9 degrees was noted, which was statistically significant (X² = 22.563; p= .003).

**Table 3:** Association of nasal septal deviation and external deformities with internal nasal valve angle (endoscopic assessment) DNS: deviated nasal septum

	Endoscopic Assessment of Angle of Internal Nasal Valve			Chi-square value
	< 9 degrees, n (%)	9-15 degree, n (%)	>15 degree, n (%)	
DNS (26)	12 (46.2%)	9 (34.6%)	5 (19.2%)	X^2^ = 22.563 p=.003
DNS with caudal dislocation (27)	25 (92.6%)	1 (3.7%)	1 (3.7%)	
DNS with spur (13)	12 (92.3%)	0 (0%)	1 (7.7%)	
DNS with External deformity (8)	6 (75%)	1 (12.5%)	1 (12.5%)	
DNS with Septal thickening (1)	1 (100%)	0 (0.0%)	0 (0.0%)	

**Figure 2:**
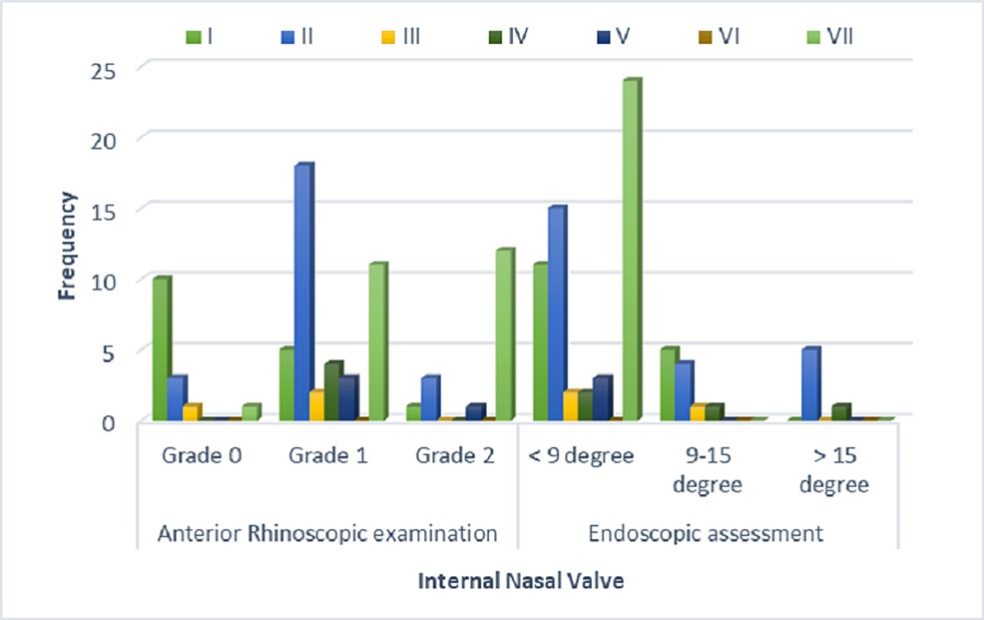
Association of Mladina classification with internal nasal valve

Table 4 and Figure 2 show the association of Mladina classification with INV on anterior rhinoscopic examination. We observed that 10 patients of Grade 0 INV (62.5%) were most common in Type 1, while 12 patients of Grade 2 INV (50.0%) were most common in Type VII. Furthermore, Type II to V had a higher prevalence of Grade 1 INV on anterior rhinoscopy examination. Thus, a linear relationship, i.e., Grade 0 INV in Type 1, Grade 1 INV in II, III, IV, and V, and Grade 2 in Type VII was observed in our study. This was found to be statistically significant. (X² = 32.791; p= .001).

**Table 4:** Association of Mladina classification with internal nasal valve (anterior rhinoscopic examination)

Mladina Classification	Anterior Rhinoscopic Examination of Internal Nasal Valve			X^2^	p-value
	Grade 0	Grade 1	Grade 2		
I (16)	10 (62.5%)	5 (31.2%)	1 (6.3%)	32.791	.001
II (24)	3(12.5%)	18(75%)	3(12.5%)		
III (3)	1(33.3%)	2 (66.7%)	0 (0.0%)		
IV (4)	0 (0.0%)	4 (100.0%)	0 (0.0%)		
V (4)	0 (0.0%)	3 (75.0%)	1 (25.0%)		
VI (0)	0(0.0%)	0(0.0%)	0(0.0%)		
VII (24)	1 (4.2%)	11 (45.8%)	12 (50.0%)		

Table 5 and Figure 2 show the association of Mladina classification with INV angle on endoscopic assessment. The important observation in the study was that all the patients with Mladina Type VII showed INV angle < 9 degrees in endoscopic assessment.

**Table 5:** Association of Mladina classification with internal nasal valve angle (endoscopic assessment) INV: internal nasal valve

Mladina Classification	Endoscopic assessment of angle of INV			X^2^	p-value
	< 9 degrees, n (%)	9-15 degree, n (%)	>15 degree, n (%)		
I (16)	11 (68.8%)	5 (31.2%)	0 (0.0%)	15.550	0.113
II (24)	15 (62.5%)	4 (16.7%)	5 (20.8%)		
III (3)	2 (66.7%)	1 (33.3%)	0 (0.0%)		
IV (4)	2 (50%)	1 (25.0%)	1 (25.0%)		
V (4)	3 (75%)	0 (0.0%)	0 (0.0%)		
VI (0)	0 (0.0%)	0 (0.0%)	0 (0.0%)		
VII (24)	24(100%)	0 (0.0%)	0 (0.0%)		

## Discussion

Patel et al. did a prospective study on patients who underwent primary external functional septorhinoplasty and studied the improvement in grades of INV postoperatively and found it to be significant [5]. We extended this approach in our study to learn about the change in grades of INV with various NSDs and external deformities. On anterior rhinoscopy examination, INV Grade 1 was found to be the most common with 18 DNS patients (69.2%), 15 DNS patients with caudal dislocation (55.6%), five DNS patients with spur (38.5%), and four DNS with external nasal deformity (50%). In DNS with caudal dislocation, DNS with spur, and DNS with external deformity, the next frequently observed grade of INV on the anterior rhinoscopy examination was Grade 2.

The angle of the INV was extensively studied endoscopically by Miman et al., questioning some dogmas regarding INV in the literature imposing the normal angle of INV value as 10-15 degrees [15]. It was observed in our study that the INV angle in endoscopic examination less than 9 degrees were observed in 25 (92.6%) DNS patients with caudal dislocation and 12 (46.2%) DNS patients. In the majority of patients in all types of NSDs with or without external nasal deformity, INV (angle) of less than 9 degrees was noted, which was statistically significant (X² = 22.563; p= .003). Yazici et al. studied the angle of INV by computerized tomography of the paranasal sinus (CT PNS) in patients undergoing septoplasty [16]. They found a significant association between the site of NSD to INV. It was found to be less than 9 degrees in most patients with NSD, with a mean angle of left INV in left-sided DNS as 8.15, and a mean angle of right INV in right-sided DNS as 8.36. Our findings of a sizeable number of patients having INV angle < 9 degrees, 56 (74.7%) DNS patients with or without external deformity, finds similarity with the above study.

In association with the Mladina classification of NSDs, we observed Grade 0 INV was most common in Type 1 (62.5%) while Grade 2 INV was most common in Type VII. Type II-V Grade 1 INV on anterior rhinoscopy examination was more common. Thus linear relationship ie., Grade 0 INV in Type 1, Grade 1 INV in types II, III, IV, and V, and Grade 2 in Type VII was observed in our study. This is found to be statistically significant (X² = 32.791; p= .001). Another important observation in the study was all the patients with Mladina Type VII had INV angle < 9 degrees in endoscopic assessment. In both the tools of assessment of INV, i.e. anterior rhinoscopic examination and endoscopic assessment, we observed Type VII was most common in Grade 2 (anterior rhinoscopic examination) and less than 9 degrees (endoscopic assessment), thus establishing a positive and complimentary role of both these assessment tools.

Study limitations

The study included only a small sample size of patients with DNS. It was not a multicentric study.

## Conclusions

The purpose of this study was to assess the INV in anterior rhinoscopy and endoscopy and its association with various NSDs with or without external nasal deformities. Anterior rhinoscopy examination of INV showed Grade 1 as the most common, with 18 patients of DNS (69.2%). In patients with DNS with caudal dislocation, DNS with spur, and DNS with external deformity, the grade of INV on the anterior rhinoscopy examination was Grade 2. On the endoscopic assessment of the INV (angle), we observed that the majority of patients, i.e., 56 patients (74.7%), had an angle less than 9 degrees. It was also noted that the majority of patients with DNS with caudal dislocation (92.6%), DNS (46.2%), DNS with spur (92.3%), and DNS with external deformity (75%) had less than 9 degrees, which was statistically significant.

A linear relationship, Grade 0 INV in Type 1, Grade 1 INV in types II, III, IV, and V, and Grade 2 in Type VII, were observed in our study, which was statistically significant. We observed Type VII was most common in Grade 2 (anterior rhinoscopic examination) and less than 9 degrees (endoscopic assessment), thus establishing a positive and complimentary role of both these assessment tools. Hence, the proposed novel classification of the angle of the INV by endoscopic assessment gives a better insight into the association of INV with various NSDs with or without external nasal deformities.
